# Roles of Phytochemicals in Cancer Prevention and Therapeutics

**DOI:** 10.3390/ijms25105450

**Published:** 2024-05-17

**Authors:** Daniel Gabriel Pons

**Affiliations:** 1 Grupo Multidisciplinar de Oncología Traslacional, Instituto Universitario de Investigación en Ciencias de la Salud (IUNICS), Universitat de les Illes Balears, Ctra. de Valldemossa, km 7.5, 07122 Palma, Illes Balears, Spain; d.pons@uib.es; 2 Grupo Multidisciplinar de Oncología Traslacional, Instituto de Investigación Sanitaria Illes Balears (IdISBa), 07120 Palma, Illes Balears, Spain

This Special Issue focused on the importance of phytochemicals for their use in the prevention and treatment of cancer. The impact of publications about the effects of phytochemicals on mammal cells has grown in the last two decades due to their antioxidant, anti-inflammatory, and antimicrobial properties at low or moderate concentrations. However, there is some controversy about the effects of phytochemicals on cancer cells at these concentration levels. In fact, the mentioned properties could help cancer cells survive, conferring resistance to chemotherapeutic agents and, consequently, cell death. On the other hand, high concentrations of these compounds can trigger drastic changes in cancer cells’ physiology, directly affecting their cell viability. Considering all these facts, the study of the roles of phytochemicals in cancer prevention and therapeutics is of great interest because of the dual effects they may have on both normal and cancer cells, depending, as well, on their concentration. In this sense, the first published article of this Special Issue was a study of the effects of high concentrations of genistein, an isoflavone mainly found in soybeans [1], on the viability of colon cancer cells depending on the modulation of oxidative stress and inflammation [2]. Previous studies in breast cancer have demonstrated that genistein, at physiological concentrations, could regulate the estrogenic response by acting as a phytoestrogen, affecting mitochondrial functionality and, therefore, inflammation, oxidative stress, and cell proliferation in breast cancer cell lines with different amounts of the estrogen receptors α and β [3,4,5]. Moreover, the phytoestrogen genistein is able to modulate the effects of chemotherapeutic agents on breast cancer cells, modulating mitochondrial functionality and depending on the estrogen receptor ratio [6]. Other phytochemicals have shown different effects on mitochondrial-related parameters, indicating the importance of this organelle in the hallmarks of cancer [7,8,9,10]. There are studies indicating that the accumulation of high concentrations of genistein in certain areas of the colon mucosa could be related to its effects on colon cells [11]. Alorda-Clara et al. have demonstrated a relationship between high concentrations of genistein treatment and a decrease in cell viability through modulation of mitochondrial biogenesis, oxidative stress, and the inflammatory status of colon cancer cells [2].

Interestingly, the other five original articles published in this Special Issue have focused on different phytochemicals and their effects on different cancer types. Augustynowicz et al. studied the anticancer potential effects of rare *Potentilla* species extracts containing phenolic, tannin, and flavonoid compounds on colon cells [12]. Some of the extracts showed anticancer properties, damaging colon cancer cell membranes, but did not reveal any cytotoxic effect against colon epithelial cells. The same authors have reported similar results, demonstrating that all *Potentilla* species may be useful sources for anticancer agents against colon tumors [13].

More concretely, Ma et al. have studied the effects of mulberry Diels-Alder-type adducts (MDAAs), and specifically the Kuwanon M (KWM) from the root bark, on apoptosis and paraptosis of lung cancer cells, associated with endoplasmic reticulum stress [14]. KWM reduced cell proliferation and migration and, at the same time, increased apoptosis through the mitochondrial pathway and paraptosis through an increment in cytoplasmic vacuolation and ER stress in A549 and NCI-H292 lung cancer cells [14]. In another study, MDAAs showed that their anticancer effects increase cell apoptosis [15], but remarkably, Ma et al. determined that KWM could affect mitochondria directly, corroborating the importance of this organelle in the response to phytochemical treatment in cancer cells [14].

In addition, the other three original research papers published in this Special Issue have studied different classical phytochemicals such as caffeine, butein, and a complex quercetin-zinc(II). Eguchi et al. analyzed the increment of anticancer drug toxicity by caffeine in a spheroid model of human lung adenocarcinoma through the reduction of the protein expression of Claudin-2 and Nrf2, affecting mitochondrial respiration and ROS production [16]. Previous studies have demonstrated the relationship between oxidative stress and Nrf2 signaling, which is linked to increased chemoresistance [17]. The results confirmed the exaggeration of doxorubicin and cisplatin toxicity mediated by caffeine treatment in these spheroids [16]. On the other hand, Park et al. revealed that butein, a flavonoid identified from *Butea monosperma*, inhibited cell growth by blocking IL-6/IL-6Rα interaction and by regulating the IL-6/STAT3/FoxO3a pathway in human ovarian cancer cells through the higher binding affinity of butien to IL-6 [18]. The results showed a decrease in cell proliferation, migration, and invasion, as well as an increase in cell cycle arrest and apoptosis [18]. Moreover, butein caused a reduction in the tumor growth of ovarian cancer cells in mouse xenografts [18]. Many drugs were found to inhibit IL-6 signaling, but none of them had promising outcomes against ovarian cancer [19]. Park et al. have found an alternative treatment for ovarian cancer through this IL-6 pathway-inhibiting mechanism [18]. Finally, Nakamura et al. studied the apoptosis induction in hepatocellular and colorectal adenocarcinoma cell lines mediated by a novel quercetin-zinc(II) complex [20]. The main results they obtained were enhanced absorption of the complex (improved bioavailability and intracellular uptake) and an increase in the anticancer efficacy with an increment of the apoptosis levels comparing the complex with the separate compounds [20]. These results agree with others demonstrating that flavonoid metal complexes penetrate lipid bilayers through hydrophobic protein pores, increasing intracellular uptake of these complexes [21].

In this Special Issue, there were published two interesting reviews about the effects of phytochemicals in cancer prevention and treatment. One of them, carried out by Na et al., shows the importance of isothiocyanates, phytochemicals present in cruciferous vegetables, in cancer prevention and therapy. Thus, the authors split the mechanisms of isothiocyanates in cancer prevention and therapy into the following four main parts: 1. regulation of microbial homeostasis in the intestinal mucosa; 2. rearrangement of energy metabolism phenotype, with a special importance of mitochondria; 3. reconstruction of tumor microenvironment, with emphasis on inflammation status; and 4. inhibition of cancer stem cells [22]. This exciting review relates the main studies of the effects of isothiocyanates in breast, liver, gastric, bladder, prostate, lung, pancreatic, glioblastoma, endometrial, and colon cancer [23,24,25,26,27,28,29,30,31,32]. On the other hand, the other review published by Golonko et al. reveals the promising synergistic effect of different types of flavonoids in combination with anthracyclines, such as doxorubicin, daunorubicin, epirubicin, or idarubicin [33]. Anthracyclines are used in many types of cancer, including breast, lymphoma, and sarcoma [34]. The mechanism of action of anthracyclines is multifactorial, i.e., disruption of DNA integrity, binding to the cell membrane, and increasing oxidative stress by an increment in free radical production [35]. The authors of this review highlight the importance of the crosstalk between flavonoids and the molecular activity of anthracyclines, with special emphasis on the following three areas of action: 1. disruption of DNA integrity [36,37,38,39,40]; 2. modulation of antioxidant response pathways [41,42,43,44,45,46,47]; and 3. inhibition of the activity of membrane proteins responsible for the active transport of drugs and xenobiotics [48,49,50,51].

All the publications in this Special Issue highlight the importance of phytochemicals in cancer prevention and therapy. Thanks to the scientific knowledge published and reviewed in this Special Issue, we are now closer to understanding the mechanisms by which various phytochemicals can directly affect the prevention and treatment of cancer. All of this from a perspective closely related to energy metabolism and mitochondria, highlighting the role of this organelle in the response of cancer cells to anticancer treatments in combination with phytochemicals.
